# Genetic variation in wheat grain quality is associated with differences in the galactolipid content of flour and the gas bubble properties of dough liquor

**DOI:** 10.1016/j.fochx.2020.100093

**Published:** 2020-06-02

**Authors:** Byoung Min, Louise Salt, Peter Wilde, Ondrej Kosik, Kirsty Hassall, Alexandra Przewieslik-Allen, Amanda J. Burridge, Mervin Poole, John Snape, Luzie Wingen, Richard Haslam, Simon Griffiths, Peter R. Shewry

**Affiliations:** a Department of Plant Science, Rothamsted Research, West Common, Harpenden, Hertfordshire AL5 2JQ, UK; b Quadram Institute Bioscience, Institute of Food Research, Norwich Research Park, NR4 7UA, UK; c Computational and Analytical Sciences Department, Rothamsted Research, Harpenden, Hertfordshire AL5 2JQ, UK; d Life Sciences, University of Bristol, 24 Tyndall Avenue, Bristol BS8 1TQ, UK; e Heygates Ltd., Bugbrooke Mill, Bugbrooke, Northampton NN7 3QH, UK; f John Innes Centre, Norwich Research Park, Colney Lane, Norwich NR4 7UH, UK

**Keywords:** Wheat, Bread making, Dough liquor, Lipidomics, Galactolipids, QTL

## Abstract

Lipids affect the quality of wheat flour for breadmaking. One possible mechanism is stabilization of the gas cells which are formed during dough mixing and expanded during fermentation, leading to a greater loaf volume and evenness of texture. We therefore compared the lipidomic profiles of flour and dough liquor fractions (which contain surface-active components present at the gas bubble interface) from two sets of wheat lines differing in allelic variation at a QTL for loaf volume. Analyses of fractions from three field trials showed consistent increases in the contents of galactolipids (monogalactosyl diglyceride and digalactosyl diglyceride) in flour and dough liquor of the lines with the increasing (good quality) allele. Biophysical analysis showed that this was associated with greater elasticity of the dough liquor fraction. This is consistent with published studies reporting a relationship between galactolipids and breadmaking quality and suggests a mechanism of action for the QTL.

## Highlights

• A QTL for breadmaking quality is associated with more galactolipids in flours.
• Dough liquor fractions from the same flours also have higher galactolipid contents.
• The dough liquor fractions with higher galactolipids exhibit increased elasticity.
• These increases indicate a mechanism of action for the quality QTL.

## 1 Introduction

Wheat is the third most important cereal crop in terms of global production and the dominant crop and major staple food in temperate countries, where it contributes between 20% and 50% of the total calories in the human diet. The global success of wheat is partly due to its wide adaptability, providing good yields in countries ranging from Argentina to Scandinavia, and upland regions in the tropics. However, it is also due to the grain processing properties, and in particular the ability of wheat flour to be processed into bread, other baked products, pasta and noodles.

The processing properties of wheat are largely determined by the gluten proteins, which correspond to the major group of storage proteins in the starchy endosperm tissue. When flour (milled grain) is mixed with water these proteins interact to form a continuous visco-elastic network which provides the cohesion required for making pasta and noodles and enables the entrapment of carbon dioxide released by bakers’ yeast during proofing to give the light porous crumb structure of leavened bread. Because of their functional importance wheat gluten proteins have been the subject of an immense volume of research dating back to the mid-18th century (Shewry et al., 2009, Hamer et al., 2009) However, gluten proteins are not the sole determinant of processing quality and other grain components also have impacts, including starch and cell wall polysaccharides. Non-protein determinants of quality have become of increasing interest in recent years as intensive selection for protein quality in breeding programmes has resulted in optimisation of protein composition, with limited opportunity for further improvement. Furthermore, the increasing fluctuation in growing conditions associated with climate change has emphasised the importance of improving the stability of processing quality which is likely to be affected, either positively or negatively, by minor grain components as well as gluten proteins.

Although lipids are minor components of wheat grain, accounting for only 2.0–2.5% of white flour, they are known to affect the volume and texture of loaves and other baked products (MacRitchie and Gras, 1973, Pareyt et al., 2011, Pycarelle et al., 2019). These effects are not completely understood, but are thought to include indirect effects by binding to and plasticising the gluten network, and direct effects by stabilizing the structure of gas cells which are formed during dough mixing and expanded during fermentation (Köhler, 2001, Chung et al., 1978). This has been suggested to result from the presence of surface-active lipids in the air–liquid interface (Salt et al., 2018) which surrounds the gas bubbles and prevents their coalescence, although this has not been demonstrated directly (Pareyt et al., 2011) However, it has been demonstrated that bread making quality can be improved by the use of specific lipases which may generate more surface-active lipid species, particularly lyso-lipids (Gerits et al., 2014, Schaffarczyk et al., 2014, Melis et al., 2020). In addition, it has been shown recently that the interfacial properties of dough liquors from different cereals link the physical properties of the interfaces occupied by lipids or mixed lipids and proteins with their foam stabilising performance and the crumb structure of baked breads (Janssen, Wouters, Pauly, & Delcour, 2018).

Wheat flour lipids comprise a highly complex mixture of neutral lipids (free fatty acids, triacylglycerols, diacylglycerols) and polar lipids (phospholipids and glycolipids), with lipidomic profiling showing up to 100 individual molecular species. Furthermore, the lipid compositions of grain and flour vary widely between genotypes and are strongly influenced by crop nutrition (nitrogen fertilisation) (Min et al., 2017) and environmental factors (Gonzalez-Thuillier et al., 2015). Consequently, it is not possible to relate simple differences in lipid composition between grain samples to differences in processing properties.

In order to determine whether wheat lipids play a role in determining breadmaking quality we have compared allelic variation at QTLs for aspects of bread quality, based on the hypothesis that the allelic differences could result from effects of dough lipids on the interfacial properties of the liquid film surrounding the gas bubbles in dough. These lines are derived from a cross between two cultivars which have good breadmaking quality, but lack “quality-related” protein subunits. Hence, it is likely that other (non-protein) components contribute to quality. In order to eliminate the confounding effects of genetic background we have developed series of near isogenic lines with the contrasting alleles at each QTL. The lines have also been grown together in field trials for three years to reduce the effects of environment. Lipidomic and biophysical studies show that the “good quality” allele at one of these QTLs (on chromosome 7A) is associated with higher levels of galactolipids in both flour and dough liquor and increased surface activity of the latter, supporting a role of dough lipids in determining gas bubble stability and suggesting a mechanism of action of the QTL.

## 2 Materials and methods

### 2.1 Identification of QTL for breadmaking quality on chromosome 7A

A doubled haploid (DH) population of 120 lines was developed by RAGT Seeds Ltd (Ickleton, Cambridgeshire, UK) from a cross between the two good quality breadmaking wheats: Malacca × Hereward (M × H) (Millar et al., 2008). These lines were grown in replicate field trials on two sites for two years, with the samples for each year being combined for milling using a Buhler MLU laboratory mill. QTLs for breadmaking quality were identified by analysis of breads made from white flour using Spiral White mixing. This is a traditional mixing system which is used in the UK by small bakers for specialist and artisan breads and was selected because it is particularly sensitive to differences in flour quality. QTLs were determined by measuring loaf volume and the number, size and wall thickness of the gas cells using C cell analysis (Millar et al., 2008) (Table 1).

**Table 1:** The QTL identified on chromosome 7A using the Malacca × Hereward population.

Chromosome	LOD	%var	Mean	Add	Nearest. marker	Trait	Increasing allele
7A	4.2	15.4	3953	117.893	AX-95186225_7A	LoafV	H
7A	6.9	31.1	0.206	−0.062	AX-94459904_7A	CELLAL	M
7A	6.2	28.1	1.143	−0.038	AX-94459904_7A	NetCELLE	M

Abbreviations are: LOD = LOD score (log of the odds); %var, percentage of phenotypic variation explained; mean, mean value for this trait from whole population; add, additive effect (negative and positive values indicate that Malacca and Hereward respectively carry the increasing allele); nearest marker, nearest molecular marker locus to QTL peak; H, Hereward; M, Malacca. Traits: LoafV, loaf volume; CELLAL, Cell alignment; NetCELLE, Net Cell elongation

### 2.2 Development, growth and analysis of NILs

To produce NILs from the QTL selected for study a DH line carrying the appropriate H allele at this QTL was crossed with Malacca and then back-crossed with Malacca four times, using the closely linked simple sequence repeat (SSR) marker *psp3001* to select for heterozygotes. A BC4 plant was then self-pollinated and series of near-isogenic streams homozygous for the Hereward (H) and Malacca (M) alleles identified. Pure stocks of NILs were multiplied under glasshouse conditions with cross pollination eliminated by placing clear bags over emerged wheat spikes prior to anthesis.

In order to reduce confounding effects of environment, the lines were grown on the same site near Norwich (UK) in 2013 (Trial 1), 2015 (Trial 2) and 2017 (Trial 3). Sets of lines with the Hereward (7A:H, 4 lines) and Malacca (7A:M, 4 lines) alleles were bulked for each year (to reduce residual genetic heterogeneity) and milled using either a Chopin CD1 laboratory mill (Trials 1 and 2) or a Buhler ML-202 mill (Trial 3) to give white flour. Nitrogen content was determined by Dumas combustion in a Leco FP 628 combustion analyser.

### 2.3 Dough liquor (DL) extraction and preparation

Non-yeasted doughs were prepared as described previously (Salt et al., 2005, Salt et al., 2006, Salt et al., 2018), using 500 g flour and 8.3 g salt with 260 mL water (7A:H) or 265 mL water (7A:M) (the difference being based on water absorptions of 53% and 52%, respectively, determined using a Brabender Farinograph). Doughs were mixed in a domestic food mixer (Kenwood Chef, Kenwood Ltd, Havant, UK) fitted with a dough hook attachment, mixing for 4 min. After dough mixing, 65 g (approximately) dough pieces were weighed into polycarbonate ultracentrifuge bottles (38 × 102 mm) with screw-on titanium caps (Beckman Coulter, item no. 355622), and held at 30 °C (in an incubator) for 90 min. The dough was then centrifuged in a pre–warmed (30 °C) fixed-angle rotor (Beckman Coulter, type 45 Ti – item no. 339160) at 200 000×*g* for 30 min at 30 °C. After ultracentrifugation, the supernatant (dough liquor) was collected, pooled and stirred for 5 min, RT, before centrifugation at 48 000×g for 20 min at 20 °C. The clarified DL was carefully aspirated, using a 10 mL disposable syringe and a 1.2 × 40 mm needle, avoiding contamination by the TAG-rich lipid pellicle on the top of the clarified DL.

### 2.4 DL surface properties

A pendant drop technique was used to determine the surface tension and surface dilatational modulus (E) of DL. Measurements were taken using an FTA 200 pulsating drop tensiometer (First Ten Angstroms, Portsmouth, VA, USA), where a droplet suspended in air was formed at the tip of a Teflon coated needle (diameter: 1.12 mm) inside a glass cuvette. The needle was connected to a 50 µL glass syringe (Hamilton Company, Reno, NV, USA). Prior to each experiment the syringe and needle were checked for contamination of surfactants by measuring the surface tension of water (72.8 mN.m^−1^) for 10 min. The surface dilatational modulus (E) of DL was determined by capturing images of a pulsating, 8–10 μL droplet (droplet size was altered depending on ST of the DL), which were taken every second for 600 s at approximately 20 °C. The shape of the droplet in each image was analysed by fitting the experimental drop profile to the Young-Laplace capillary equation to calculate surface tension, volume and specific area. E was calculated from the amplitude of the change in surface area and change in surface tension. The surface pressure (π) was calculated by subtracting the measured surface tension from the surface tension of the water phase (72.8 mN.m^−1^).

### 2.5 Lipid extraction

Flours and dough liquors were analysed for lipid composition by ESI-MS/MS (Min et al., 2017, Gonzalez-Thuillier et al., 2015, Salt et al., 2018). Lipids were extracted from flour samples as described by Finnie, Jeannotte, and Faubion (2009). The flour (150 mg) was heated in boiling water (100 °C) for 12 min to inactivate any hydrolytic enzymes Three sequential extractions were then carried out with petroleum ether (PEt), water-saturated butan-1-ol (1:10) (WSB), and propan-2-ol/water (90:10) (IW), with sample to solvent ratios of 1:10, 1:14, and 1:10, respectively. The PEt and WSB extracts were washed by shaking with 1:1 (v/v) 0.88% KCl, centrifugation for 2 min at 650×g, and recovery of the upper layer to a new tube, in which all three lipid phases were combined. Lipids were extracted from dough liquor following the Bligh and Dyer method (Bligh & Dyer, 1959) with modifications. Chloroform: methanol (1:2) was added to 1 mL DL in a 2:7.5 ratio. Samples were vortex-mixed and incubated with agitation for 15 min, 250 rpm at room temperature. After 10 min of centrifugation at 650 g, the supernatant, containing the dough lipids, was transferred to a new tube. Lipid extraction was repeated using 3.75 mL chloroform: methanol: water (1:2:0.8). The two serial extracts were collected in the same tube. The supernatants were washed with equal parts of chloroform and 0.88% KCL, 1:3.2:3.2 sample: solvent: salt solution ratio. The lower phase was collected in a new tube after centrifugation for 5 min at 650×g. The aqueous phase was re-extracted with 2.5 mL of chloroform. For all samples, the combined extracts were evaporated under nitrogen at 40 °C, re-suspended in chloroform and filtered (0.45 μm Millex-FH filters, Merck Millipore, Germany), dried under a stream of nitrogen, re-suspended in 1 mL of chloroform, flushed with nitrogen and stored at − 80 °C.

### 2.6 Lipid analysis

Quantitative analyses of polar lipids, (phosphatidylcholine (PC), phosphatidylethanolamine (PE), phosphatidylinositol (PI), phosphatidylglycerol (PG), LPC, DGDG or MGDG) lipids were carried out using electrospray ionization tandem triple quadrupole mass spectrometry (API 4000 QTRAP; Applied Biosystems; ESI-MS/MS) as described previously by Gonzalez-Thuillier et al. (2015). The internal standards for polar lipids were supplied by Avanti (Alabama, USA), incorporated as; 8 pmol 13:0-LPC, 0.086 nmol di24:1-PC, 0.080 nmol di14:0-PE, 0.05 nmol di18:0-PI, 0.080 di14:0-PG and 0.03 nmol di18:0-PS. The standards dissolved in chloroform and different conditions were used for the aqueous samples, 100 μL foam or 25 μL un-foamed DL were combined with chloroform/methanol/300 mM ammonium acetate (300:665:3.5 v/v) to make a final volume of 1 mL. The lipid extracts were infused at 15 μL/min with an autosampler (HTS-xt PAL, CTC-PAL Analytics AG, Switzerland). Data acquisition and acyl group identification were as described by Gonzalez-Thuillier et al. (2015). The data were processed using the LipidView software (SCIEX, Framingham, MA, U.S.A.), where isotope corrections were applied. The peak area for each lipid was normalized to the internal standard and further normalized to the weight of the initial sample.

### 2.7 Genetic mapping and QTL analysis

A genetic map was constructed using the Axiom® 35 K breeders’ array genotypes scored by the Functional Genomics Group at the University of Bristol. All steps were conducted in the R software suite (vs. 3.6.1). The Malacca × Hereward linkage map was constructed using package ASMap (vs. 1.0–4) using the p-value of 10-^16 to define linkage groups. In a second round of genetic mapping, linkage groups derived from the same chromosome were attempted to be joined up using a p-value of 10^-3. Pictures of the genetic maps were plotted using package “LinkageMapView” (vs. 2.1.2) supplementary Fig. QTL detection was performed using package “qtl” (vs. 1.44–9) (Broman, Wu, Sen & Churchill, 2003) in two steps, the first scan determining co-factors and the second scan identifying robust QTL, taking the co-factors into account.

## 3 Results

### 3.1 Identification of a QTL on chromosome 7A

The Malacca × Hereward Axiom 35 K genotyping results are shown in Supporting Material Table S1 and the linkage map in Supporting Material Fig. S1. The study identified a number of QTLs for aspects of breadmaking quality, including a QTL on chromosome 7A (Table 1 and Supporting Material Fig. S2) at which the Hereward (H) allele was associated with increased loaf volume, decreased cell alignment and decreased net cell elongation (Table 1). The 7A QTL was selected for the production of sets of NILs which are referred to as 7A:H and 7A:M, respectively.

### 3.2 Lipidomic analyses of flour and dough liquor from Trials 1 and 2

Lipidomic analysis was initially used to determine the full lipid profiles (up to 40 molecular species) of white flours from the bulked sets of NILs grown in Trial 1. Five replicate extracts of each flour were analysed, showing nine species of galactolipid (GL): four species of monogalactosyl diglyceride (MGDG 34:2, 36:, 36:4 and 36:5) and five species of digalactosyl diglyceride (DGDG 34:2, 34:3, 36:3, 36:4 and 36:5). For both lipid classes the 36:4 species was dominant, and all GL species were present at significantly higher concentrations in 7A:H than in 7A:M (p-values ranging from 0.003 to 0.045) (Table 2). Fifteen molecular species of phospholipid (five species of lysophosphatidyl choline (LPC), four of phosphatidyl choline (PC), four of phosphatidyl ethanolamine (PE) and two of phosphatidyl glycerol (PG)) and ten species of free fatty acids (FFA) were also determined (Supporting Material Table S2). Only one species, FFA 22:1, differed significantly in amount between the two samples (being higher in 7A:M, p-value 0.049), but the difference was small in quantitative terms (132.94 compared with 125.59 nmol g^−1^) (Supporting Material Table S2).

**Table 2:** Contents of galactolipid species in 5 replicate samples of flours from the 7A:M and 7A:H sets of NILs grown in Trial 1.

Sample Replicates	MGDG 34:2	MGDG 36:3	MGDG 36:4	MGDG 36:5	DGDG 34:2	DGDG 34:3	DGDG 36:3	DGDG 36:4	DGDG 36:5
7A:M R1	3.51	5.02	88.29	11.43	34.93	8.06	12.3	141.85	37.07
7A:M R2	4.43	4.98	90.99	12.20	35.37	8.16	11.90	144.21	27.84
7A:M R3	6.92	7.68	222.88	29.47	44.98	10.91	14.60	188.54	38.99
7A:M R4	4.69	6.91	106.35	15.53	34.39	8.07	11.69	144.37	28.71
7A:M R5	5.11	7.98	118.89	16.93	43.99	11.32	15.58	151.10	38.57
Average	4.93	6.51	125.48	17.11	38.73	9.30	13.21	154.01	34.24
Standard Error	0.56	0.64	24.97	3.25	2.36	0.74	0.79	8.77	2.46
7A:H R1	6.54	15.13	230.81	31.43	44.80	11.13	16.32	183.67	39.90
7A:H R2	5.22	9.30	174.07	25.65	67.34	16.32	24.31	282.91	55.21
7A:H *R*3	7.62	11.34	170.70	25.29	58.09	12.99	19.58	233.14	51.00
7A:H R4	6.54	14.32	224.05	31.58	72.48	18.49	24.74	284.87	57.36
7A:H R5	6.81	18.66	242.61	33.20	76.20	17.77	23.87	306.10	63.41
Average	6.55	13.75	208.45	29.43	63.78	15.34	21.76	258.14	53.38
Standard Error	0.39	1.61	15.03	1.65	5.64	1.41	1.65	22.13	3.92
*t*-statistic	2.367	4.171	2.847	3.378	4.1	3.778	4.686	4.373	4.137
df	8	8	8	8	8	8	8	8	8
p-value	0.045	0.003	0.022	0.01	0.003	0.005	0.002	0.002	0.003

Nine species of galactolipid are quantified: four species of monogalactosyl diglyceride (MGDG 34:2, 36:3, 36:4 and 36:5) and five species of digalactosyl diglyceride (DGDG 34:2, 34:3, 36:3, 36:4 and 36:5).

Significance is assessed via a two-sample *t*-test. Where variances were deemed unequal, Satterthwaite’s approximation to the degrees of freedom was used to calculate Welch’s *t*-test.

**Fig. 1:**
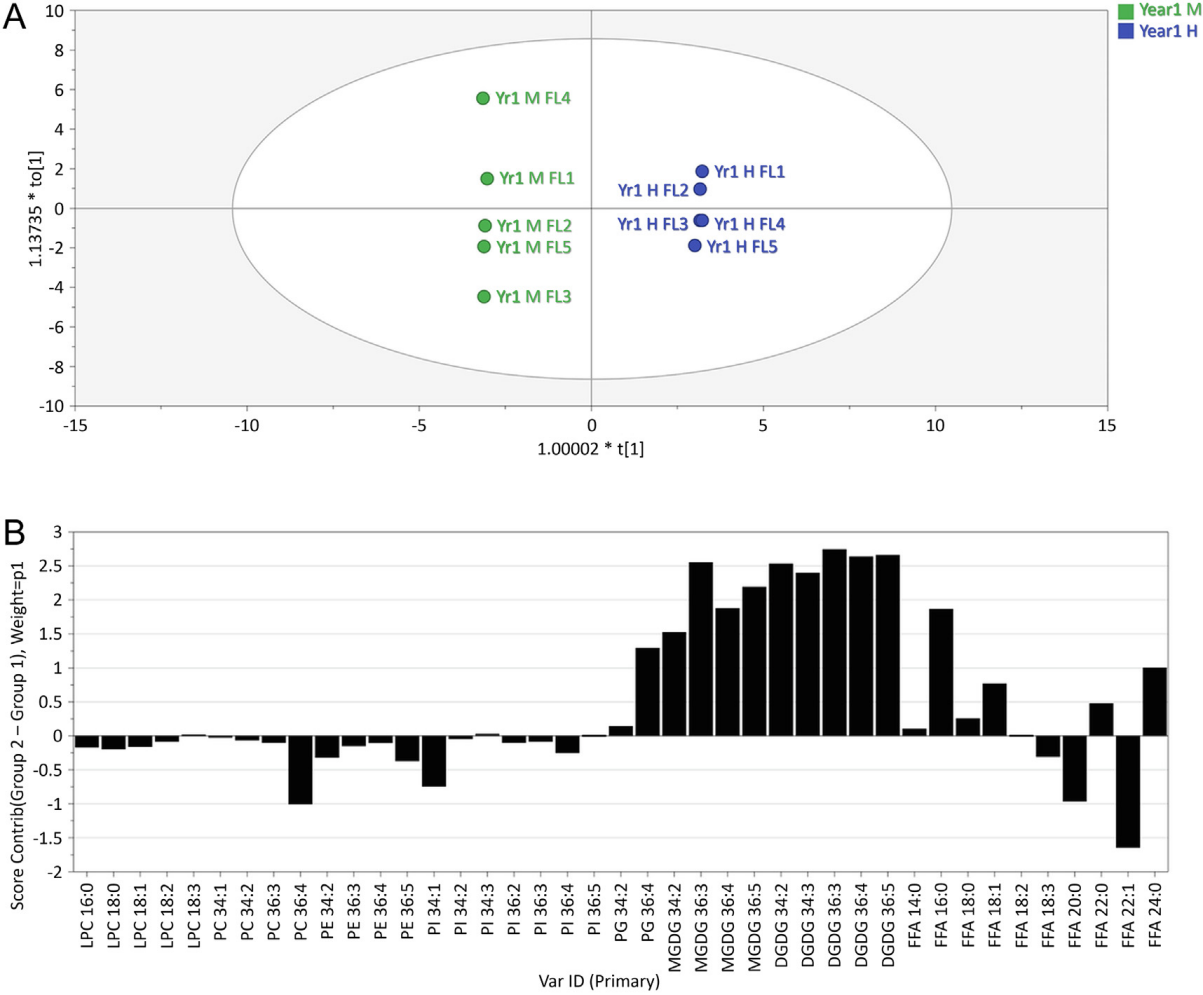
Orthogonal partial least squares discriminant analysis (OPLS-DA) of lipid profiles of 5 replicate samples of flour from lines with the 7A:M and 7A:H alleles A: Scores plot, coloured according to allele; B: Contribution plot comparing the alleles.

**Fig. 2:**
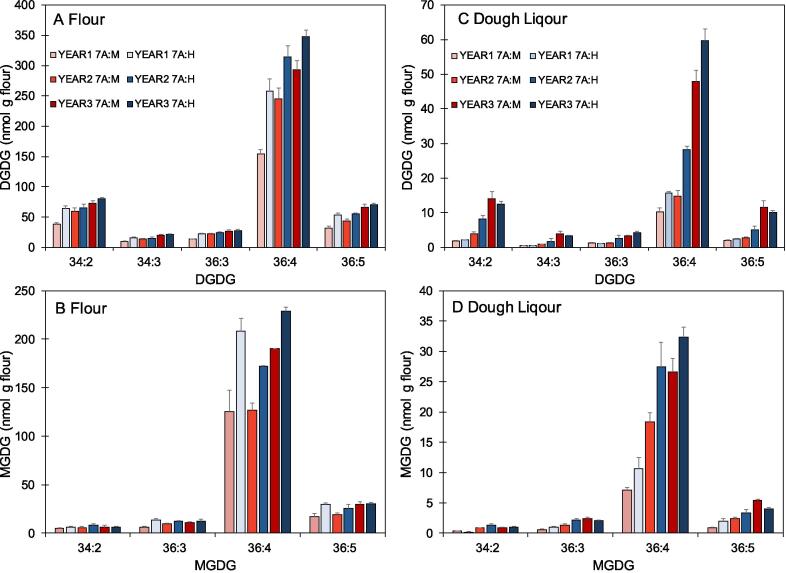
Contents of galactolipid molecular species in flour and dough liquor fractions from lines with the 7A:M and 7A:H alleles grown in three years (replicated analysis of five samples +/- standard error).

The lipid profiles were also compared using supervised multivariate analysis, orthogonal partial least squares discriminant analysis (OPLS-DA), to identify differences between the two alleles (Fig. 1). This confirmed that the 7A:H and 7A:M were clearly separated based on the GL profiles Further studies therefore focused on the amounts and properties of the GL components and the increase in GLs in 7A:H confirmed by analyses of flours from Trial 2 (Fig. 2).

Dough liquor is the aqueous fraction prepared by centrifugation of dough and is considered to contain the surface-active components present in the liquid film which lines the gas bubbles and determines their ability to expand and retain gas during proofing. We therefore determined the GL content and composition of dough liquor fractions from the set of NILs grown in Trials 1 and 2. This showed consistently higher contents of the two major GL species (MGDG36:4 and DGDG36:4) in the flours and dough liquors from 7A:H (Fig. 2).

### 3.3 Analysis of flours and dough liquors from Trial 3

More detailed studies were therefore carried out on the lines grown in Trial 3, using white flour from a laboratory scale Buhler mill (extraction rate 78%). Flours and dough liquor fractions were initially analysed as in Trials 1 and 2, confirming that the Hereward allele was associated with greater proportions of MGDG36:4 and DGDG36:4 in both fractions (Fig. 2).

**Fig. 3:**
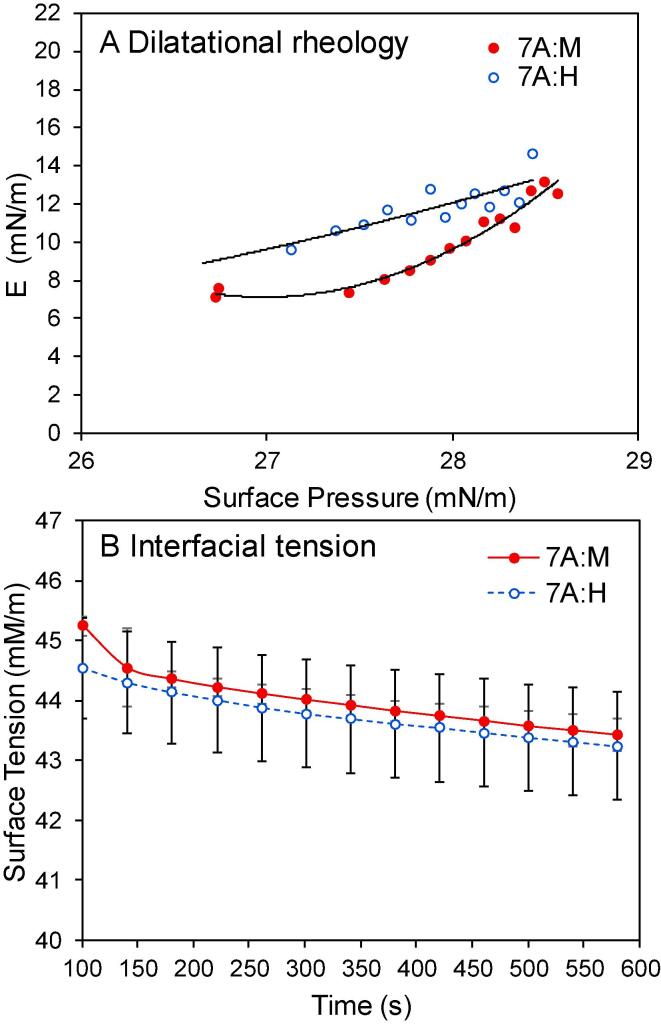
Surface properties at the air–water interface of dough liquors derived from flour from NILs 7A:M () and 7A:H (). (A) Surface dilatational modulus (E) as a function of surface pressure and (B) surface tension as a function of time.

To determine whether the differences in GL composition were associated with differences in the surface properties of the dough liquor fractions, aliquots of the fractions used for lipidomic analysis were analysed to determine their surface dilatational rheology and surface tension (Fig. 3).

Mean surface dilatational elastic modulus (E), obtained from 15 intervals over 600 s, was plotted against averaged surface pressure (π). The resulting plot is sensitive to interfacial composition and qualitatively indicates the types of molecules adsorbed at the air/water interface of DL (Fig. 3 A).

The earliest adsorption time equates to the lowest values of π, and as adsorption continues, π increases. The initial increase in π relates to the development of an adsorbed layer at the air:water interface due to the migration of surface active molecules in the DL to the interface followed by subsequent rearrangement and interaction. The π values obtained for both NILs ranged between 26 and 29 mN/m; similar to values previously determined by Salt et al. (2018) for undiluted dough liquor (from cv Hereward) that was enriched with galactolipids; specifically, DGDG. Individually, DL from 7A:H flour had π values ranging between 27.5 and 28.5 mN/m and DL from 7A:M flour had π values ranging from 26.7 to 28.5 mN/m (approximately) (Fig. 3A). The higher starting values of π for DL from 7A:M flour suggested that it adsorbed more quickly, and this was confirmed by studying the kinetics of the surface tension values (Fig. 3B) but the values between the samples were not significantly different beyond 150 s adsorption.

The low E values for DL from both NILs demonstrated relatively weak elastic properties indicating a surface that was dominated by lipids. DL from 7A:M flour produced the least elastic interface; where E ranged between 7.2 and 13.5 mN/m (approximately) and were lowest at the start of the experiment (100 s) with higher values being determined at the end of the experiment (600 s). The surface of DL from 7A:H flour gave slightly higher E values, ranging between 9.5 and 15.0 mN/m (approximately). Although E from both NILs had similar values at the end of the experimental period, the formation rate and relationship with π were slightly different, suggesting a subtle difference in the interfacial composition between the two NILs during the formation of the surface layer.

## 4 Discussion

The Malacca × Hereward population was selected to identify novel QTLs for grain processing quality because is derived from a cross between two cultivars which have good breadmaking quality but lack the “high quality” high molecular weight glutenin subunit alleles (1Ax1 or 1Ax2* and 1Dx5 + 1Dy10) which are usually present in European breadmaking cultivars (Hereward having the composition 1Ax null, 1Dx3 + 1Dy12 and 1Bx7 + 1By9 and Malacca 1Ax null, 1Dx2 + 1Dy12 and 1Bx17 + 1By18). Payne (Payne, 1987) assigned values to HMW subunit alleles to represent their relative quality, which can be summed to calculate “quality scores” for cultivars. When this is done for Hereward and Malacca their quality scores are 4 and 5, respectively, compared with a maximum score of 10 (in which good quality subunits are encoded by all three genomes). It is therefore reasonable to assume that the good breadmaking quality of these two cultivars is related to components other than HMW subunits of glutenin, whether proteins or other components. We therefore developed a high-density genetic linkage map to facilitate the dissection of quality traits in this population, leading to the identification of a novel QTL for bread making quality on chromosome 7A. In order to avoid the conflicting effects of variation in genetic background between the cultivars and the DH lines with the Hereward and Malacca alleles at the 7A QTL, the trait was “Mendelianised” by back-crossing into Malacca followed by segregation of the two alleles in a common background.

Lipids are minor components of wheat grain, accounting for only 2.0–2.5% dry wt. of white flour, with galactolipids accounting for up to 18% of total lipids (on a mol % basis) in white flour fractions (Gonzalez-Thuillier et al., 2015). Similarly, lipids only account for about 15% of the dry weight of dough liquor, the main components being proteins and carbohydrates (authors’ unpublished data). Nevertheless, lipids are known to affect the volume and texture of loaves (MacRitchie & Gras, 1973) and the interfacial properties of the dough liquor fraction which corresponds to the liquid lining the gas bubbles in expanded dough (Salt et al., 2018). This is mainly because lipids, particularly soluble polar lipids and surfactants, are far more surface active than proteins and polymers on a molar basis. They can close pack at the interface giving rise to much lower interfacial tensions than proteins, thus can outcompete proteins for interfacial areas, even at relatively low concentrations (Wilde, Mackie, Husband, Gunning, & Morris, 2004). In particular, galactolipids have been reported to improve bread-making performance by stabilising the liquid film lamellae (dough liquor) at the gas cell interface (Selmair & Koehler, 2009). Consistent with this, lipase treatments which improve dough quality have been suggested to improve gas retention by effects on the surface properties of the dough liquor fraction (Primo-Martin, Hamer, & de Jongh, 2006), with enzymes which convert MGDG and DGDG to their monoacyl products (MGMG and DGMG, respectively) being particularly effective (Schaffarczyk et al., 2014). Hence, it was logical to focus on lipids, and particularly surface-active components present in the dough liquor fraction, in the present study.

However, the lipid content and composition of flour and dough liquor are also known to be highly affected by the environment (Salt et al., 2018). It was therefore necessary to determine the compositions of flour and dough liquor from grain samples grown in the field over several seasons. Despite substantial differences in lipid composition between the years, the differences between the alleles in the compositions of both the flours and dough liquors were statistically significant and consistent over the three years, with no similar differences being observed for other lipid species (out of over 40 quantified).

Our previous comparison of flours from three years (Salt et al., 2018) showed significant differences in π and E between samples, which correlated with significant differences in galactolipid (GL), phospholipid (PL) and free fatty acid (FFA) compositions in the dough liquor. The increased GL and PL together with decreased FFA concentrations resulted in improved foam stability and increased loaf volume correlated with a more rapid development of surface elasticity of the DL (Salt et al., 2018).

The DL from both NILs displayed low E values, showing relatively weak elastic properties and indicating a surface that was dominated by lipids, as the interfacial elasticity of proteins present in dough liquor is generally much higher than that of lipids, and is therefore a strong indicator of which species is dominant at the interface (Salt et al., 2006). This agrees with previous measurements on DL (Salt et al., 2006, Salt et al., 2018). Although the differences in lipid composition between the NILs in the present study are smaller than in our previous study (Salt et al., 2018), it is well known that even relatively small amounts of lipids can have a significant effect on surface rheology in mixed protein lipid systems (Wilde, 2000, Wilde et al., 2004), including in dough liquor (Salt et al., 2006). The 7A:H allele had consistently higher levels of MGDG 36:4 and DGDG 36:4 in the DL than 7A:M allele. The more rapid development of E for the 7A:H allele is consistent with our previous study which showed that increased GL concentrations in both DL and the foam correlated with the more rapid development of an elastic interface, better foam stability and greater loaf volume (Salt et al., 2018). It is also consistent with the greater dough strength determined by rheological analysis as the gas volume can influence dough strength (Chin, Martin & Campbell 2005). The properties, size and number of bubbles will contribute to the overall dough strength. This could be due to gas bubbles with higher surface elasticities being less deformable and thus contribute to the overall rheology of the dough.

The results therefore indicate that the greater dough strength associated with the Hereward allele at the 7A QTL results from increased elasticity at the gas bubble interface due to higher contents of surface-active galactolipids. The molecular marker information presented here will facilitate the selection of this QTL in breeding.

## CRediT authorship contribution statement

**Byoung Min:** Conceptualization, Investigation, Formal analysis, Writing - original draft. **Louise Salt:** Investigation, Writing - review & editing. **Peter Wilde:** Conceptualization, Supervision, Writing - original draft, Writing - review & editing. **Ondrej Kosik:** Formal analysis. **Kirsty Hassall:** Formal analysis. **Alexandra Przewieslik-Allen:** Investigation, Formal analysis. **Amanda J. Burridge:** Investigation, Formal analysis. **Mervin Poole:** Conceptualization, Supervision, Writing - original draft, Writing - review & editing. **John Snape:** Conceptualization, Supervision. **Luzie Wingen:** Conceptualization, Formal analysis, Visualization, Writing - review & editing. **Richard Haslam:** Conceptualization, Supervision, Investigation, Formal analysis, Visualization, Writing - original draft, Writing - review & editing. **Simon Griffiths:** Conceptualization, Formal analysis, Supervision, Writing - original draft, Writing - review & editing. **Peter R. Shewry:** Conceptualization, Supervision, Writing - original draft, Writing - review & editing, Project administration, Funding acquisition.

## Declaration of Competing Interest

The authors declare that they have no known competing financial interests or personal relationships that could have appeared to influence the work reported in this paper.
