# Supplementary figures and images for: Genetic variation in wheat grain quality is associated with differences in the galactolipid content of flour and the gas bubble properties of dough liquor
Source: Food Chem X. 2020 Jun 2;6:100093. doi: 10.1016/j.fochx.2020.100093 (PMC7292906; doi:10.1016/j.fochx.2020.100093)

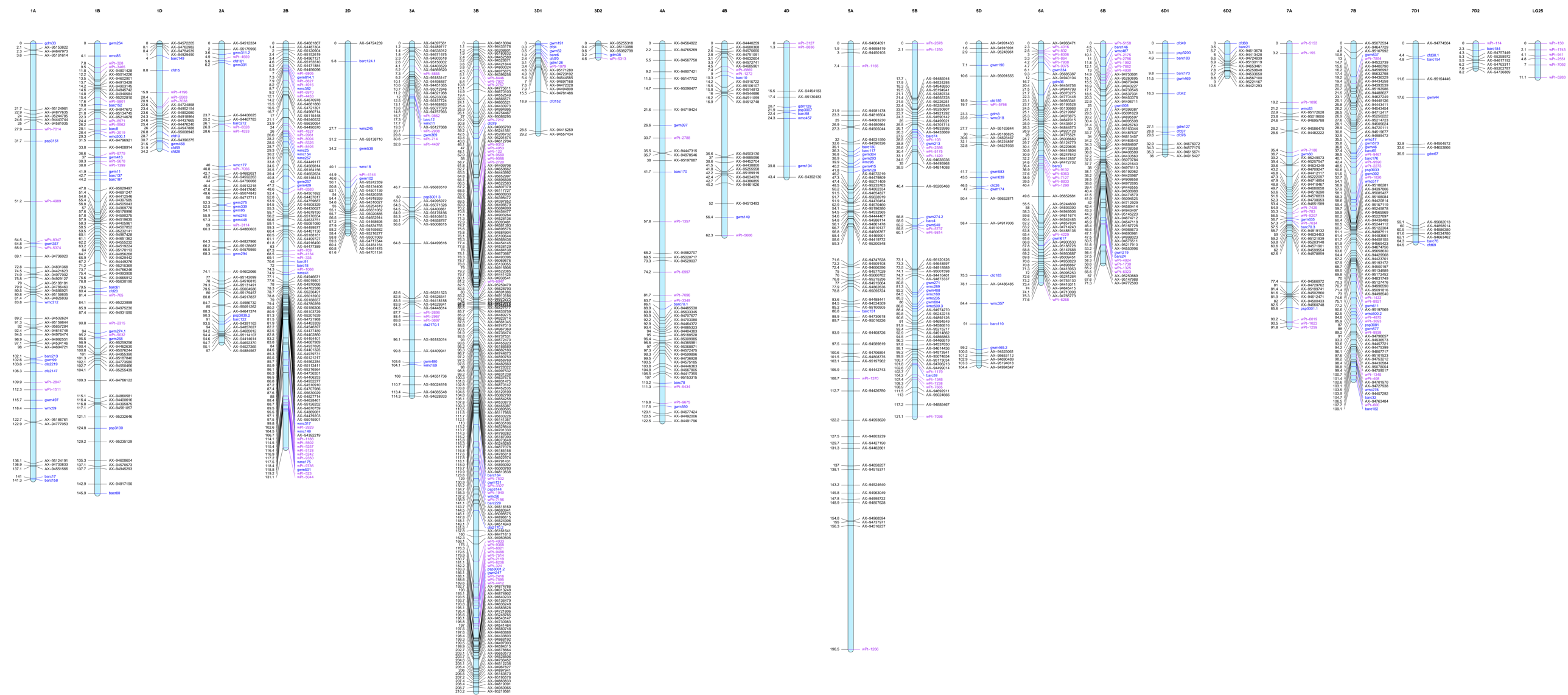

Supplement: Supplementary data 1 [file mmc1.pdf]
