# Supplementary material for: Genetic variation in wheat grain quality is associated with differences in the galactolipid content of flour and the gas bubble properties of dough liquor
Source: Food Chem X. 2020 Jun 2;6:100093. doi: 10.1016/j.fochx.2020.100093 (PMC7292906; doi:10.1016/j.fochx.2020.100093)

Figure S2. Malacca x Hereward 7A QTL plots.

Traits: LoafV , loaf volume; CELLAL , Cell alignment; NetCELLE , Net Cell elongation. Horizontal axis represents genetic linkage map of 7A short arm on left, long arm on right. Dotted horizontal line shows LOD significance threshold. Horizontal bar with circle shows 1 LOD confidence interval with the circle representing QTL peak marker. QTL peak markers shown above peak in red text.

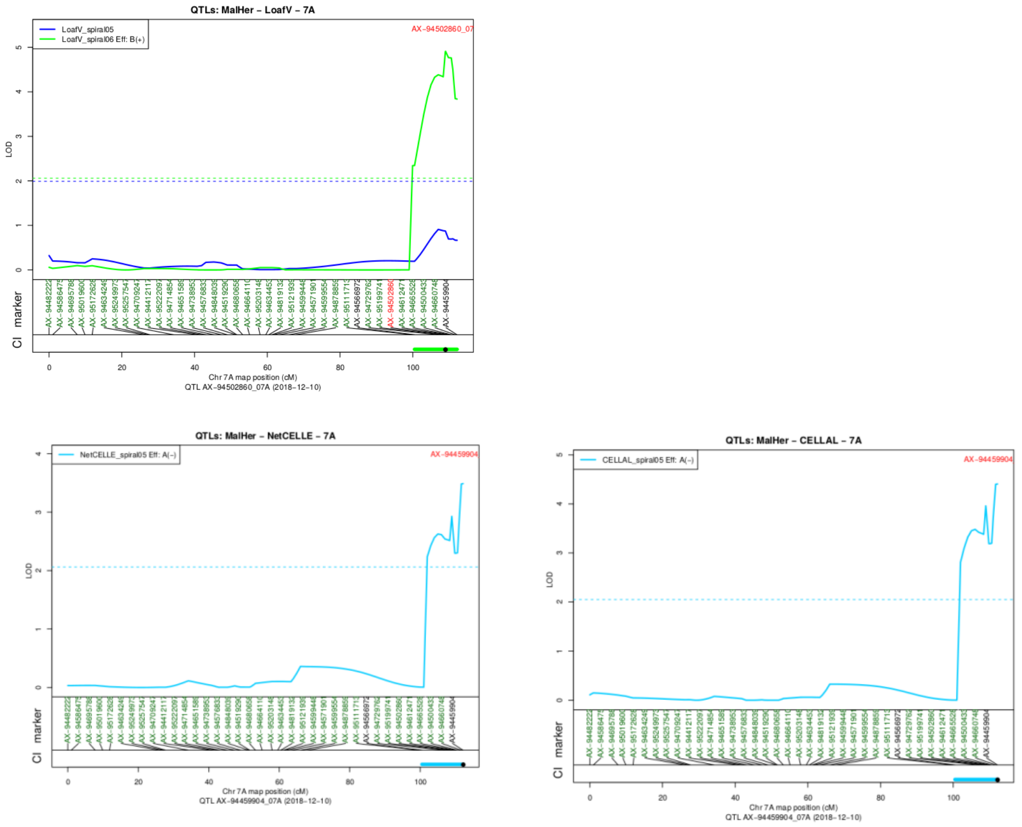

Supplement: Supplementary data 2 [file mmc2.pdf]
