# Supplementary material for: Genetic variation in wheat grain quality is associated with differences in the galactolipid content of flour and the gas bubble properties of dough liquor
Source: Food Chem X. 2020 Jun 2;6:100093. doi: 10.1016/j.fochx.2020.100093 (PMC7292906; doi:10.1016/j.fochx.2020.100093)
Supplement: Supplementary data 4 [file mmc4.docx]

Table S2. Contents of phospholipid species and free fatty acids in 5 replicate samples of flour from the 7AM and 7AH sets of NILs grown in Trial 1.

LPC, lysophosphatidyl choline, PC, phosphatidyl choline, PE, phosphatidyl ethanolamine, PG, phosphatidyl glycerol.

Significance was assessed via a two-sample t-test. Where variances were deemed unequal, Satterthwaite’s approximation to the degrees of freedom was used to calculate Welch’s t-test.

|  | **Phospholipid (nmol mg^-1^)** | | | | | | | | | | | | |  |  |
| --- | --- | --- | --- | --- | --- | --- | --- | --- | --- | --- | --- | --- | --- | --- | --- |
| **Sample Replicates** | **LPC 16:0** | **LPC 18:0** | **LPC 18:1** | **LPC 18:2** | **LPC 18:3** | **PC 34:1** | **PC 34:2** | **PC 36:3** | **PC 36:4** | **PE 34:2** | **PE 36:3** | **PE 36:4** | **PE 36:5** | **PG**  **34:2** | **PG**  **34:3** |
| 7 AM R1 | 1744.76 | 54.15 | 175.25 | 1729.62 | 115.14 | 13.54 | 113.83 | 22.95 | 85.65 | 4.70 | 1.21 | 8.51 | 1.75 | 28.78 | 10.59 |
| 7 AM R2 | 1543.32 | 39.71 | 168.07 | 1607.77 | 119.39 | 14.56 | 112.33 | 20.29 | 99.26 | 4.39 | 1.50 | 10.22 | 1.22 | 23.20 | 13.00 |
| 7 AM R3 | 1518.35 | 40.80 | nd | 1425.85 | 105.06 | 15.11 | 93.74 | 18.30 | 80.36 | 8.54 | 2.27 | 21.45 | 1.72 | 39.45 | 14.88 |
| 7 AM R4 | 2219.25 | 64.46 | 279.19 | 2377.05 | 143.88 | 19.58 | 128.87 | 31.35 | 120.65 | 6.00 | 3.20 | 13.02 | 2.69 | 30.58 | 9.20 |
| 7 AM R5 | 1629.73 | 32.40 | 151.40 | 1525.61 | 88.46 | 25.73 | 155.90 | 38.59 | 144.84 | 5.61 | 2.08 | 14.40 | 1.43 | 22.42 | 11.24 |
| **Average** | 1731.08 | 46.30 | 193.48 | 1733.18 | 114.39 | 17.70 | 120.93 | 26.30 | 106.15 | 5.85 | 2.05 | 13.52 | 1.76 | 28.89 | 11.78 |
| **Standard Error** | 128.31 | 5.74 | 29.00 | 168.50 | 9.10 | 2.26 | 10.37 | 3.79 | 11.92 | 0.73 | 0.34 | 2.23 | 0.25 | 3.07 | 0.99 |
| 7 AH R1 | 1673.60 | 43.87 | 186.17 | 1653.53 | 126.66 | 11.57 | 75.81 | 16.41 | 61.48 | 3.32 | 1.71 | 8.23 | 1.02 | 32.76 | 13.97 |
| 7 AH R2 | 1768.66 | 52.27 | nd | 1872.74 | 139.12 | 15.82 | 99.76 | 19.75 | 42.65 | 4.28 | 1.56 | 9.56 | 1.25 | 22.10 | 14.75 |
| 7 AH R3 | 1733.54 | 40.96 | 128.13 | 1648.12 | 115.74 | 15.86 | 97.49 | 18.44 | 94.95 | 6.30 | 1.93 | 14.81 | 1.45 | 36.38 | 13.82 |
| 7 AH R4 | 1474.45 | 43.23 | 188.45 | 1614.32 | 101.56 | 20.91 | 136.90 | 29.83 | 72.36 | 6.70 | 2.24 | 15.83 | 2.27 | 35.60 | 13.73 |
| 7 AH R5 | 1577.68 | 27.63 | 155.88 | 1585.33 | 100.68 | 20.80 | 160.07 | 34.72 | 113.00 | 4.44 | 1.78 | 12.88 | 1.26 | 29.16 | 13.12 |
| **Average** | 1645.59 | 41.59 | 164.66 | 1674.81 | 116.75 | 16.99 | 114.01 | 23.83 | 76.89 | 5.01 | 1.84 | 12.26 | 1.45 | 31.20 | 13.88 |
| **Standard Error** | 53.65 | 3.98 | 14.26 | 50.99 | 7.38 | 1.76 | 15.13 | 3.57 | 12.37 | 0.64 | 0.12 | 1.47 | 0.22 | 2.60 | 0.26 |
| **t-statistic** | -0.615 | -0.675 | -0.892 | -0.332 | 0.202 | -0.249 | -0.378 | -0.473 | -1.704 | -0.862 | -0.572 | -0.47 | -0.941 | 0.575 | 2.054 |
| **df** | 8 | 8 | 6 | 4.727 | 8 | 8 | 8 | 8 | 8 | 8 | 8 | 8 | 8 | 8 | 4.56 |
| **p-value** | 0.556 | 0.519 | 0.407 | 0.754 | 0.845 | 0.81 | 0.716 | 0.649 | 0.127 | 0.414 | 0.583 | 0.651 | 0.374 | 0.581 | 0.101 |

| **Free Fatty Acids (nmol mg^-1^)** | | | | | | | | | | |
| --- | --- | --- | --- | --- | --- | --- | --- | --- | --- | --- |
| **Sample Replicates** | **FFA 14:0** | **FFA 16:0** | **FFA 18:0** | **FFA 18:1** | **FFA 18:2** | **FFA 18:3** | **FFA 20:0** | **FFA 22:0** | **FFA 22:1** | **FFA 24:0** |
| 7 AM R1 | 124.34 | 6994.70 | 246.38 | 340.63 | 1428.46 | 163.24 | 153.88 | 159.91 | 137.77 | 124.56 |
| 7 AM R2 | 126.83 | 1173.96 | 287.97 | 180.47 | 1557.97 | 175.75 | nd | 154.70 | 127.70 | 138.10 |
| 7 AM R3 | 123.91 | 1044.52 | 211.22 | 215.96 | 1461.23 | 154.38 | 141.25 | 164.79 | 131.66 | 134.49 |
| 7 AM R4 | 126.83 | 675.46 | 258.38 | 306.00 | 1581.55 | 228.83 | 175.17 | 169.87 | 130.37 | 125.63 |
| 7 AM R5 | 135.16 | 1207.00 | 298.61 | 287.81 | 1448.08 | 187.66 | 145.49 | 138.06 | 137.25 | 126.30 |
| **Average** | 127.41 | 2219.13 | 260.51 | 266.17 | 1495.46 | 181.97 | 153.95 | 157.47 | 132.95 | 129.82 |
| **Standard Error** | 2.03 | 1197.61 | 15.56 | 29.55 | 31.00 | 13.00 | 6.75 | 5.47 | 1.97 | 2.72 |
| 7 AH R1 | 130.81 | 1240.69 | 342.76 | 380.80 | 1566.92 | 197.57 | 146.43 | 154.26 | 124.44 | 134.93 |
| 7 AH R2 | 127.41 | 1273.37 | 341.51 | 205.12 | 1579.17 | 156.42 | 131.37 | 168.11 | 119.65 | 141.92 |
| 7 AH R3 | 120.63 | 1426.15 | 193.80 | 379.48 | 1566.05 | 141.08 | 137.63 | 171.30 | 126.94 | 136.26 |
| 7 AH R4 | nd | 1320.34 | 241.43 | 430.66 | 1580.63 | 197.09 | 137.45 | 168.91 | nd | 137.64 |
| 7 AH R5 | 137.71 | 1236.20 | 310.83 | 212.34 | 1214.39 | 140.26 | 151.71 | 154.11 | 131.34 | 128.31 |
| **Average** | 129.14 | 1299.35 | 286.07 | 321.68 | 1501.43 | 166.48 | 140.92 | 163.34 | 125.59 | 135.81 |
| **Standard Error** | 3.18 | 35.09 | 29.50 | 47.04 | 71.82 | 12.92 | 3.61 | 3.77 | 2.18 | 2.21 |
| **t-statistic** | 0.445 | -0.768 | 0.766 | 0.999 | 0.076 | -0.845 | -1.673 | 0.884 | -2.377 | 1.71 |
| **df** | 7 | 4.007 | 8 | 8 | 8 | 8 | 7 | 8 | 7 | 8 |
| **p-value** | 0.67 | 0.485 | 0.466 | 0.347 | 0.941 | 0.423 | 0.138 | 0.402 | 0.049 | 0.126 |
